# MARK/Par1 Kinase Is Activated Downstream of NMDA Receptors through a PKA-Dependent Mechanism

**DOI:** 10.1371/journal.pone.0124816

**Published:** 2015-05-01

**Authors:** Laura P. Bernard, Huaye Zhang

**Affiliations:** Department of Neuroscience and Cell Biology, Robert Wood Johnson Medical School, Rutgers, the State University of New Jersey, Piscataway, New Jersey, United States of America; Centre national de la recherche scientifique, University of Bordeaux, FRANCE

## Abstract

The Par1 kinases, also known as microtubule affinity-regulating kinases (MARKs), are important for the establishment of cell polarity from worms to mammals. Dysregulation of these kinases has been implicated in autism, Alzheimer’s disease and cancer. Despite their important function in health and disease, it has been unclear how the activity of MARK/Par1 is regulated by signals from cell surface receptors. Here we show that MARK/Par1 is activated downstream of NMDA receptors in primary hippocampal neurons. Further, we show that this activation is dependent on protein kinase A (PKA), through the phosphorylation of Ser431 of Par4/LKB1, the major upstream kinase of MARK/Par1. Together, our data reveal a novel mechanism by which MARK/Par1 is activated at the neuronal synapse.

## Introduction

The microtubule affinity-regulating kinase (MARK)/partitioning-defective 1 (Par1) family of Ser/Thr kinases plays an essential role in various cellular processes including the establishment of cell polarity, cell cycle regulation, vesicular transport and cell migration [1,2]. There are four members of the mammalian MARK/Par1 family, MARK1-4. These four members are highly homologous in their catalytic domain (100% homologous) and more variable in their C-terminal spacer domain (73% homologous) [3]. A number of substrates for MARKs have been identified, which include microtubule-associated proteins (MAPs), Cdc25, doublecortin, HDAC7 and PSD-95 [1,4]. Despite an important role for MARK/Par1 in different cellular processes, it remains unclear how MARK/Par1 activity is regulated by signals from cell surface receptors.

We and others have recently shown that MARK/Par1 is important for the morphogenesis of dendritic spines in hippocampal neurons [4,5,6]. Dendritic spines are tiny protrusions that receive most of the excitatory synaptic inputs in the mammalian brain. Spines undergo dynamic changes in their number, size and shape in response to synaptic activity [7]. This activity-driven structural and functional plasticity is dependent on the NMDA subtype of glutamate receptors [8,9], and is believed to be important for cognitive processes such as learning and memory [10,11,12,13]. NMDA receptors are calcium-permeable ionotropic glutamate receptors. NMDA receptor stimulation and the subsequent calcium-influx lead to the activation of many downstream effectors proteins, such as the Calcium/calmodulin dependent protein kinase II (CaMKII) [14], PI3 kinase [15,16], ERK [17,18], and protein kinase A (PKA) [8,19,20,21,22].

Here we report that the MARK/Par1 kinases are activated downstream of NMDA receptors. Further, we elucidate the mechanism by which NMDA receptors activate MARK/Par1. We show that Par1 activation is not dependent on several known effectors of NMDA receptors, such as CaMKII, PI3 kinase, or ERK. Instead, Par1 activation is dependent on PKA. Finally, we show that Par1 is activated by PKA-dependent phosphorylation of Par4/LKB1 on Ser431. Taken together, our studies reveal a novel mechanism for MARK/Par1 activation by NMDA receptors.

## Materials and Methods

### Antibodies and reagents

For Western blot analyses, the following primary antibodies were used: phospho-MARK family (Thermo Scientific, Cat. No. PA5-17495, 1:1000); MARK1/Par1c (ProteinTech, Cat. No. 21552-1-AP, 1:1000); phospho-LKB1 (Santa Cruz, Cat. No. sc-28465-R, 1:2000); LKB1 (Santa Cruz, Cat. No. sc32245, 1:2000); α-tubulin (DSHB, Cat. No. AA4.3, 1:4000); GAPDH (Millipore, Cat No. MAB374, 1:5000). Secondary antibodies used include Horseradish peroxidase conjugated goat anti-rabbit and goat anti-mouse antibodies (Jackson ImmunoResearch, 1:10,000), Alexa 488-conjugated goat anti-rabbit and Alexa 594-conjugated goat anti-mouse antibodies (Invitrogen, 1:1,000). For pharmacological treatments of neurons, the following reagents were used: picrotoxin (Sigma, 10μM final); bicuculline (Tocris, 40μM final); TTX (Tocris, 1μM final); CNQX (Tocris, 10μM final); NMDA (Sigma, 50μM final); EGTA (Sigma, 2mM final); APV (Sigma, 100μM final); 4-AP(Sigma, 1mM final); H-89 (LC laboratories,10μM final); forskolin (LC laboratories, 0.1–10μM final).

### Hippocampal neuron culture

Hippocampal neuron cultures were prepared as described previously [4,23]. Briefly, hippocampi from E18 Sprague-Dawley rats were incubated in 0.25% trypsin for 15 min at 37°C, washed, and triturated using a fire-polished Pasteur pipette. Neurons were plated on 35 mm tissue culture dishes coated with 0.1mg/ml poly-L-lysine (Sigma Cat. No. P2636). Cultures were maintained in Neurobasal medium supplemented with B27 and 2mM GlutaMax. On DIV3, cytosine arabinoside (araC) was added to a final concentration of 5μM.

### Pharmacological treatments and western blotting

Hippocampal neurons were used for experiments at DIV11-13. For NMDA stimulation, neurons were pretreated with CNQX and TTX for 30 min, then stimulated with 50 μM NMDA for 5 min. For treatment with kinase inhibitors, the inhibitor was included in the pretreatment as well as during the 5 min NMDA stimulation. For stimulation of synaptic NMDA receptors, hippocampal neurons were treated with 4-AP and PTX, or 4-AP and bicuculline, for 10 min. Neurons were then lysed in RIPA buffer containing 20mM Hepes, 150mM NaCl, 0.5% NP-40, 1% Triton X-100, 0.25% deoxycholate, 2mM EDTA, 2mM EGTA, 10mM DTT, supplemented with protease inhibitor cocktail (Sigma P-8340, 1:1000), phosphatase inhibitor cocktail (Sigma P-0044, 1:100), 1mM PMSF, 10mM β-glycerophosphate, 10mM NaF. Lysates were cleared by centrifugation at 14,000xg for 10 min at 4°C. For isolation of the synaptosomal fraction, neurons were homogenized in buffer containing 0.32M sucrose, 10mM Hepes, pH 7.4, supplemented with the above-mentioned protease and phosphatase inhibitors. Homogenates were centrifuged at 1,000xg for 10 min at 4°C. The resulting supernatant was centrifuged at 17,500xg for 30 min at 4°C, and the pellet (crude synaptosomal fraction) was resuspended in RIPA buffer, centrifuged again at 17,500xg for 30 min and the supernatant was used for subsequent analysis. Lysates were separated by SDS-PAGE, transferred to PVDF membrane and probed by the indicated antibodies. Proteins were visualized by enhanced chemiluminescence (ECL) and imaged using Syngene G:Box/iChemi-XR and the GeneSnap software (Version 7.09.a) (Syngene, Frederick, MD).

### Immunocytochemistry and imaging

Hippocampal neurons (DIV24) grown on coverslips were fixed and permeabilized in 100% MeOH for 20 min at -20°C. Neurons were then blocked with 20% goat serum in PBS for 1 hour at room temperature, and incubated with primary antibodies diluted in 5% goat serum overnight at 4°C. After three washes in PBS, neurons were incubated with Alexa fluorophore-conjugated secondary antibodies for 1 hour at room temperature. They were then washed again with PBS for three times, and mounted on slides with VectaShield mounting media. Confocal images were taken with an Olympus FV1000 microscope using a 60x water immersion objective.

### Quantification and statistical analysis

Blots were background subtracted and quantified using NIH ImageJ. Phospho-Par1 signal in each sample was measured and normalized to the Par1 signal. The p-Par1/Par1 ratio of each condition is then normalized to the control condition. Similarly, the p-LKB1/LKB1 ratio was measured and normalized to the control condition. Paired t-test was used to determine statistical significance. Data are expressed as Mean ± SD.

### Ethics statement

Procedures involving vertebrate animals were approved by the Institutional Animal Care and Use Committee (IACUC) of Rutgers Robert Wood Johnson Medical School (Protocol # I13-055-10). Pregnant rats were euthanized by isoflurane overdose followed by pneumothorax. Embryonic tissue was then harvested.

## Results

**Fig 1:**
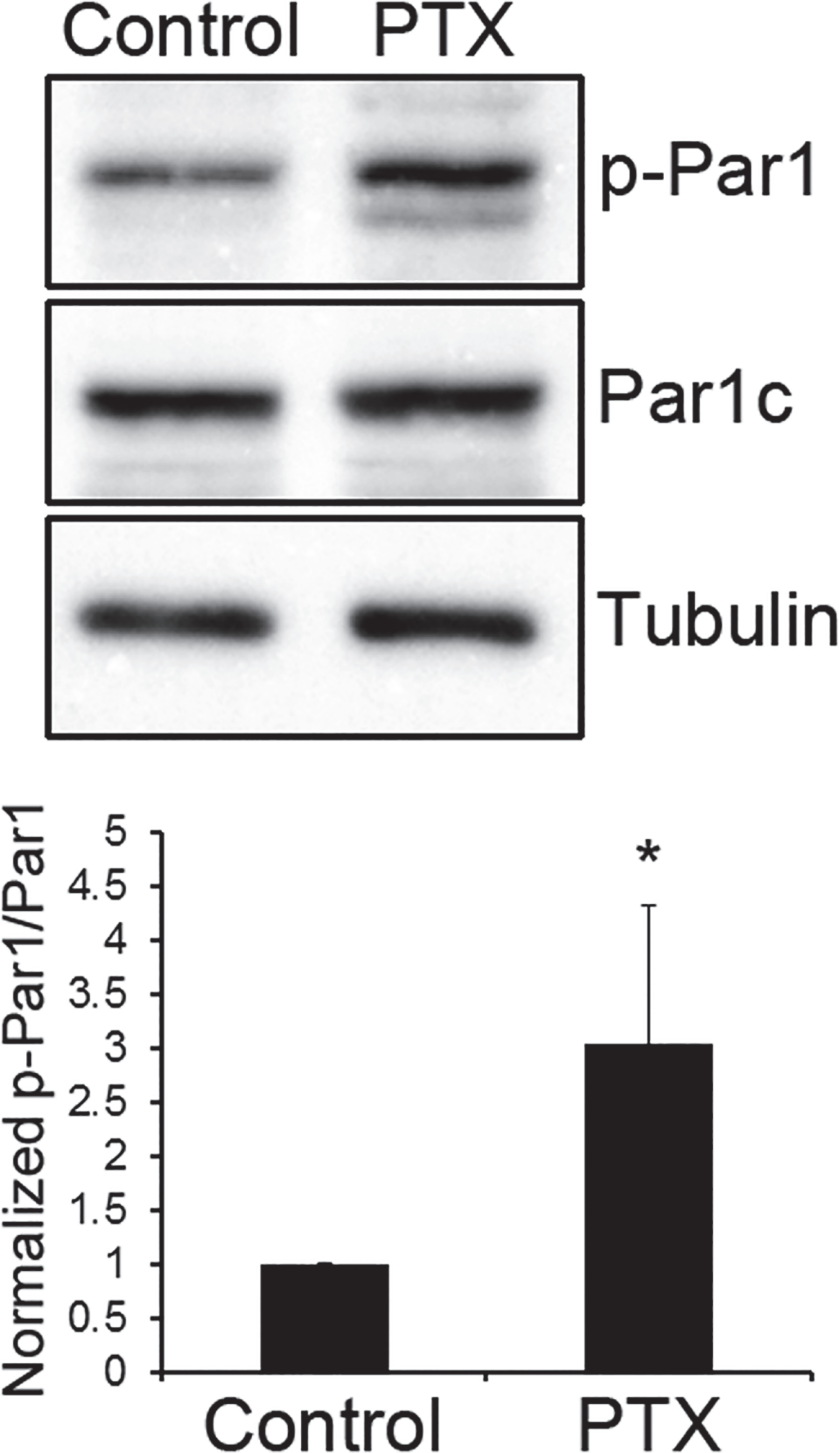
MARK/Par1 is regulated by synaptic activity. Hippocampal neurons were treated with either DMSO (Control) or 10 μM of picrotoxin (PTX) for one hour. After treatment, neurons were lysed and immunoblotted with the indicated antibodies. Representative blots are shown in (a) and quantifications are shown in (b), expressed as Mean ± SD. *p<0.01, n = 5.

We recently showed that the polarity protein Par1 plays an important role in dendritic spine morphogenesis by phosphorylating the synaptic scaffolding protein PSD-95 on Ser561 [4]. However, it remained unclear whether and how Par1 is regulated by synaptic activity. To see if Par1 is regulated by synaptic activity, we first treated cultured hippocampal neurons with picrotoxin (PTX). PTX inhibits γ-aminobutyric acid type A (GABA_A_) receptors. Thus, it enhances the endogenous excitatory synaptic activity in the neuronal network. As seen in Fig 1, compared with control hippocampal neurons treated DMSO, neurons treated with picrotoxin exhibited an increase in Par1 activation (p<0.01, n = 5), as shown by a phospho-specific antibody against the activation loop threonine of MARK/Par1 family [24]. The observed increase in Par1 activity was most prominent during the period of rapid synaptogenesis in cultured neurons (S1 Fig) [25,26], and the synaptosomal fraction showed a similar increase in Par1 activity (S2 Fig). This suggests that Par1 kinase activity is regulated by synaptic activity.

**Fig 2:**
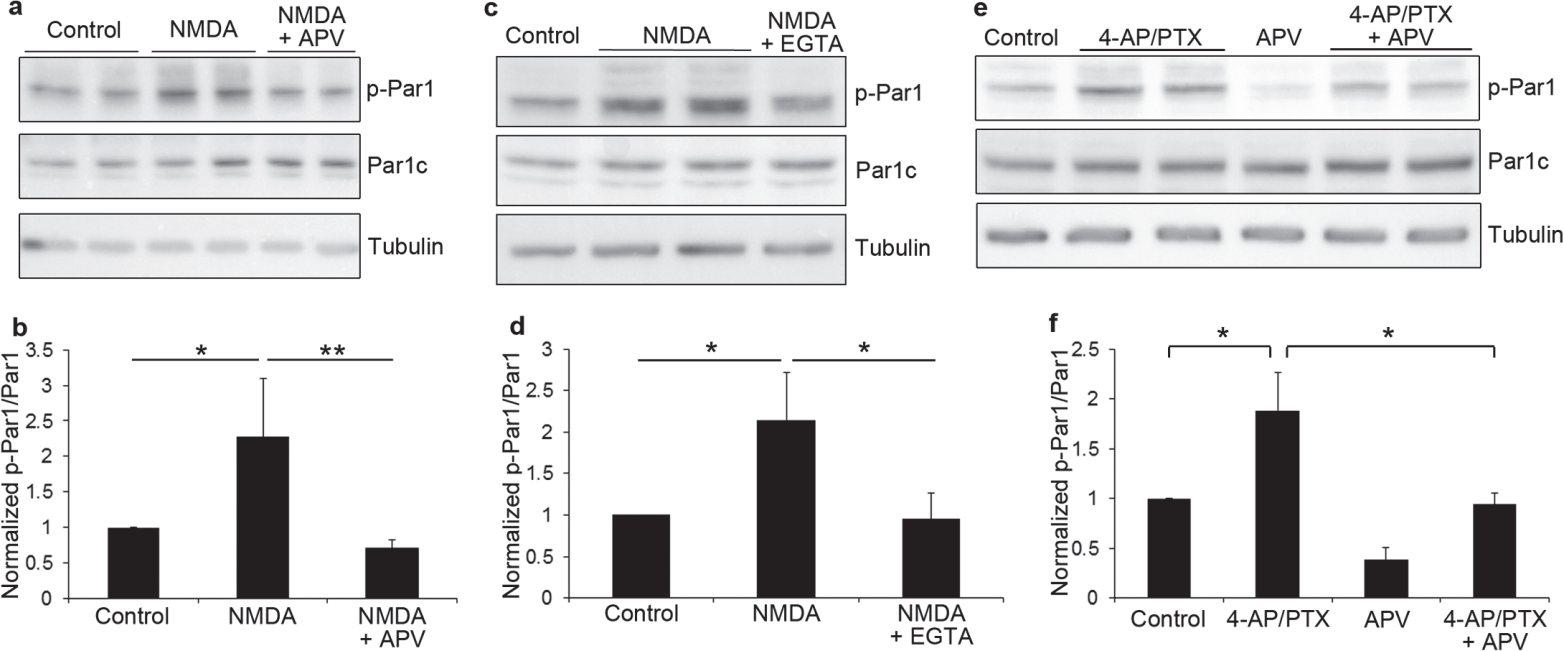
MARK/Par1 is activated by NMDA receptors. a. Hippocampal neurons were pretreated with CNQX and TTX for 30 minutes, then stimulated with 50 μM of NMDA for 5 min. For inhibition of NMDA receptors, APV was included in the pretreatment and during NMDA stimulation. Neurons were lysed and immunoblotted with the indicated antibodies. b. Quantifications of blots in (a). Data are Mean ± SD, *p<0.05, **p<0.01, n = 4. c. Hippocampal neurons were pretreated with CNQX and TTX for 30 minutes, then stimulated with 50 μM of NMDA for 5 min. For EGTA treatment, EGTA was included in the pretreatment and during NMDA stimulation. Neurons were lysed and immunoblotted with the indicated antibodies. d. Quantifications of blots in (c). Data are Mean ± SD, *p<0.01, n = 4. e. Hippocampal neurons were treated with 4-AP and picrotoxin for 10 minutes. For inhibition of NMDA receptors, APV was included in the pretreatment and during 4-AP/PTX stimulation. Neurons were lysed and immunoblotted with the indicated antibodies. f. Quantifications of blots in (e). Data are Mean ± SD, *p<0.005, n = 5.

To identify the specific receptors mediating the activation, we treated neurons with agonists for AMPA or NMDA receptors. Treatment of hippocampal neurons with AMPA did not lead to any significant changes in Par1 activity (data not shown). By contrast, a 5 minute treatment with NMDA led to a significant increase in Par1 activity. This increase was reversed by the NMDA receptor antagonist APV (Fig 2A and 2B, *p<0.05, **p<0.01, n = 4), as well as EGTA (Fig 2C and 2D, *p<0.01, n = 4), which suggests that NMDA receptor-mediated calcium influx is necessary for Par1 activation.

To further confirm these results, we used an alternative method of stimulation. It is known that synaptic NMDA receptors can be activated by treating neurons with a combination of a potassium channel antagonist and a GABA_A_ receptor antagonist [27,28,29]. Thus, we stimulated hippocampal neurons with PTX and 4-aminopyridine (4-AP), a potassium channel antagonist. Similar to NMDA treatment, we observed a significant increase in Par1 activity using the 4-AP/PTX treatment (Fig 2E and 2F, *p<0.005, n = 5). Similar results were obtained with 4-AP and bicuculline, another GABA_A_ receptor antagonist (S3 Fig). The increase is also reversed by APV suggesting the observed activation is NMDA receptor-dependent.

**Fig 3:**
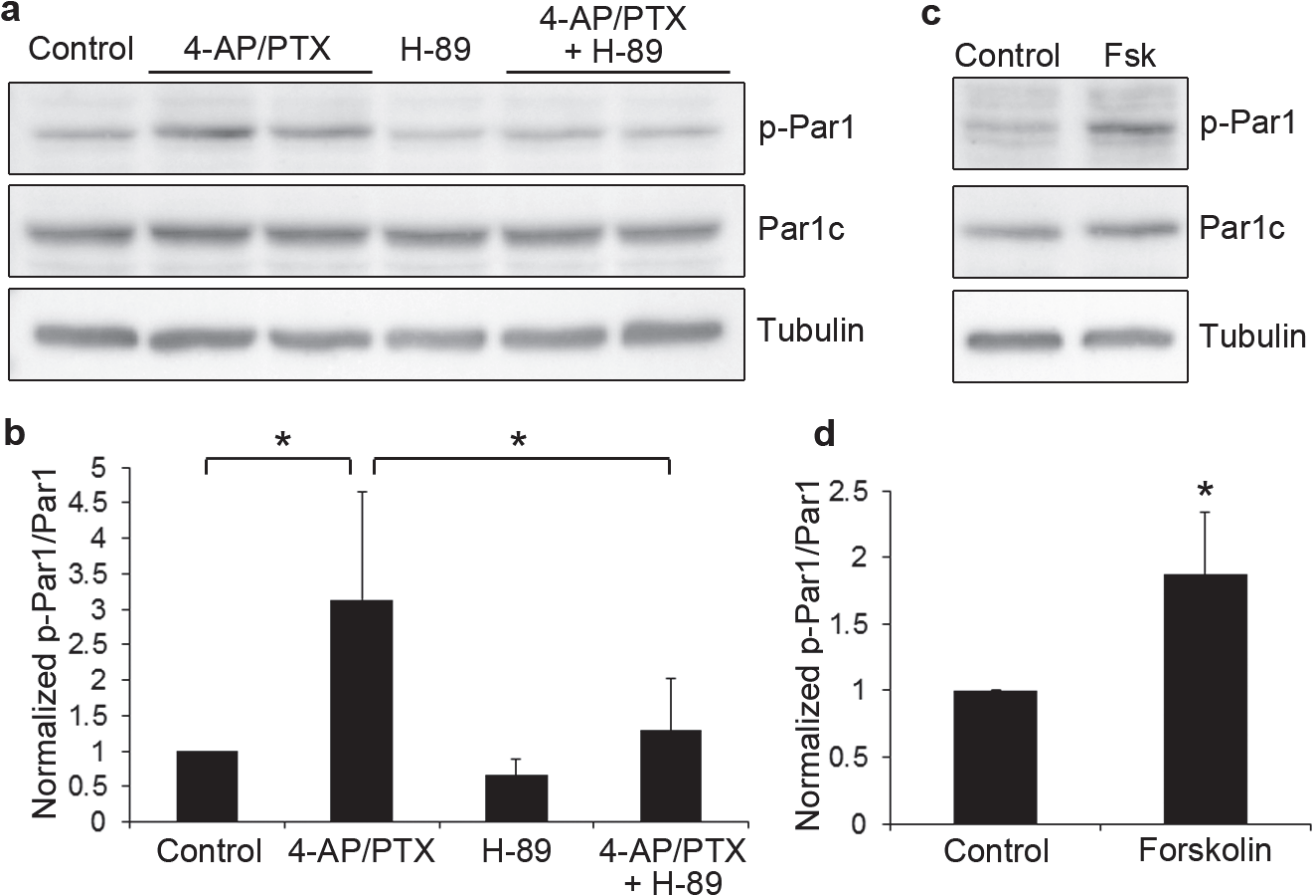
MARK/Par1 is activated by NMDA receptors through PKA. a. Hippocampal neurons were treated with 4-AP and picrotoxin for 10 minutes. For inhibition of PKA, H-89 was included in the pretreatment and during 4-AP/PTX stimulation. Neurons were lysed and immunoblotted with the indicated antibodies. b. Quantifications of blots in (a). Data are Mean ± SD, *p<0.01, n = 5. c. Hippocampal neurons were treated with DMSO (Control) or 10 μM forskolin (Fsk) for 10 minutes. Neurons were lysed and immunoblotted with the indicated antibodies. d. Quantifications of blots in (c). Data are Mean ± SD, *p<0.01, n = 4.

Our next goal was to determine the mechanism by which NMDA receptors activate Par1. To examine this, we applied different inhibitors of kinases known to be downstream of NMDA receptors. Inhibitors to CaMKK (STO-609), CaMKII (KN62), PI3K (Wortmannin and LY294002), or ERK (U0126) did not reverse the Par1 activation (Table 1); however, H-89, an inhibitor to PKA, efficiently reversed the NMDAR-induced Par1 activation (Fig 3A and 3B, *p<0.01, n = 5). Consistent with this observation, forskolin, an activator of PKA, induced Par1 activation (Fig 3C and 3D, *p<0.01, n = 4). These results suggest that Par1 is activated downstream of NMDA receptors through a PKA-dependent mechanism.

**Table 1:** Effects of different inhibitors on NMDA-induced MARK/Par1 activation.

Inhibitor	NMDA stimulation Fold increase of p-Par1/Par1 over control (Mean ± SD)	NMDA + inhibitor Fold increase of p-Par1/Par1 over control (Mean ± SD)	p value
KN62	2.15 ± 0.21	2.01 ± 0.30	0.45
STO609	2.20 ± 0.74	2.03 ± 0.84	0.85
Wortmannin	2.24 ± 0.29	2.02 ± 0.11	0.42
LY294002	2.05 ± 0.72	1.85 ± 0.53	0.72
U0126	2.68 ± 0.38	2.87 ± 0.46	0.69

Hippocampal neurons were pretreated with CNQX and TTX for 30 minutes, then stimulated with 50 μM of NMDA for 5 min (NMDA stimulation). For treatment of different inhibitors (NMDA + inhibitor), the indicated inhibitor was included in the pretreatment and during NMDA stimulation. Neurons were lysed and immunoblotted for phospho-Par1, Par1 and tubulin. Blots were quantified by measuring the phospho-Par1 band intensity and normalizing to the intensity of the Par1 bands.

**Fig 4:**
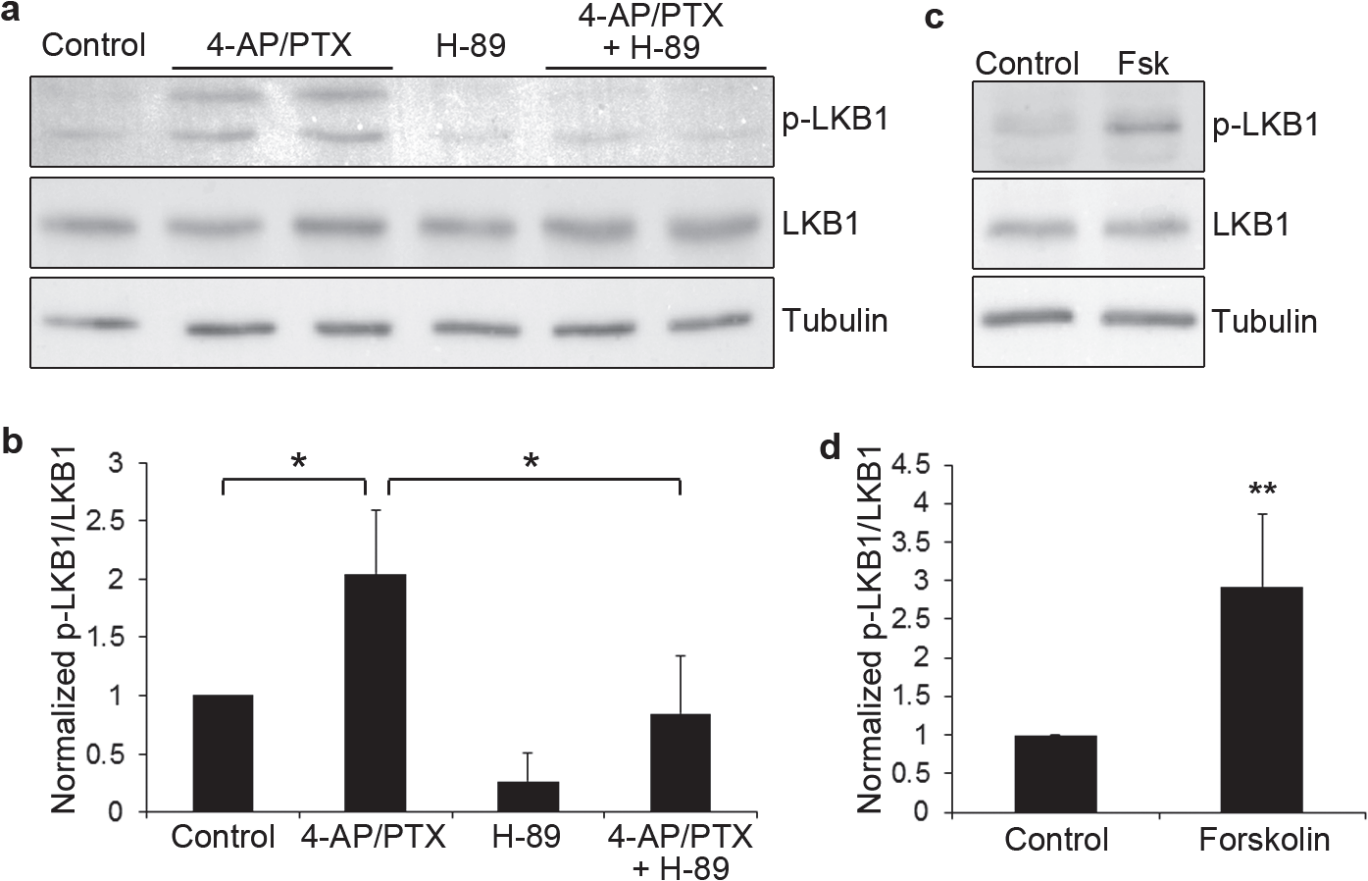
Par4/LKB1 is activated by NMDA receptors through PKA. a. Hippocampal neurons were treated with 4-AP and picrotoxin for 10 minutes. For inhibition of PKA, H-89 was included in the pretreatment and during 4-AP/PTX stimulation. Neurons were lysed and immunoblotted with the indicated antibodies. b. Quantifications of blots in (a). Data are Mean ± SD, *p<0.01, n = 4. c. Hippocampal neurons were treated with DMSO (Control) or 10 μM forskolin (Fsk) for 10 minutes. Neurons were lysed and immunoblotted with the indicated antibodies. d. Quantifications of blots in (c). Data are Mean ± SD, *p<0.001, n = 5.

Since PKA is known to phosphorylate Ser431 of Par4, also known as LKB1, which the major upstream activator of Par1 [1,30,31,32], we hypothesized that NMDA receptors activate Par1 through PKA and Par4/LKB1. To test this hypothesis, we examined Ser431 phosphorylation of Par4/LKB1. NMDA receptor activation induced a significant increase in Ser431 phosphorylation of Par4/LKB1, as shown by a phospho-specific antibody against the Ser431 site of LKB1 [33]. This increase is reversed by the PKA inhibitor H-89 (Fig 4A and 4B, *p<0.01, n = 4). Conversely, forskolin treatment induces Par4/LKB1 phosphorylation at Ser431 in a dose-dependent manner (Fig 4C and 4D, *p<0.001, n = 5, and S4 Fig). This shows that Par4/LKB1 is activated downstream of NMDA receptors through PKA-dependent phosphorylation of Ser431. Moreover, we found that phospho-Ser431 LKB1 was present at excitatory synapses (S5 Fig) suggesting that this pathway is activated at the synapses. Taken together, our results show that Par1 is activated downstream of NMDA receptors, through PKA-dependent phosphorylation of Par4/LKB1.

## Discussion

Recent studies from us and others show an important role for the MARK/Par1 family of kinases in dendritic spine morphogenesis in hippocampal neurons [4,5,6]. However, it is unclear whether and how Par1 is regulated by synaptic activity. Here we show that Par1 is activated downstream of NMDA receptors. Further, we show that this activation is dependent on PKA-mediated phosphorylation of Ser431 of Par4/LKB1, which is the major upstream kinase of Par1. Since PKA is activated downstream of adenylyl cyclase (AC)-mediated increase in cAMP [34], and AC is activated by NMDA receptor-mediated calcium influx [35], our studies elucidate an NMDA receptor-PKA-LKB1-Par1 pathway that mediates the activation of the Par1 kinases at synapses.

Since the activation of NMDA receptor is essential for synaptic plasticity, our results raise the exciting possibility that NMDA receptor-mediated Par1 activation is involved in activity-dependent remodeling of synapses. Our recent studies show that Par1 phosphorylates the synaptic scaffolding protein PSD-95 on Ser561 [4]. PSD-95 is known to be involved in activity-dependent synaptic plasticity [36]. Overexpression of PSD-95 blocks long-term potentiation while facilitating long-term depression [37,38]. In addition, PSD-95 knockout mice have a number of behavioral problems including impaired learning and drug addiction [39,40]. Upon plasticity-inducing stimuli, the postsynaptic density (PSD) undergoes active remodeling. PSD-95 is known to transiently dissociate from the PSD during this process and then subsequently re-stabilizes [41]. It will be of great interest to examine whether Par1-mediated phosphorylation of PSD-95 plays a role in this NMDA receptor-dependent PSD-95 dynamics.

Moreover, Par1 is known to regulate microtubule dynamics through phosphorylating microtubule-associated proteins like MAP2 and tau [1]. In hippocampal neurons, knockdown of MARK2/Par1b results in decreased microtubule growth and a reduction in spine accumulation of p140Cap [5], a scaffolding protein important for normal spine morphology and synaptic plasticity [42,43]. Since dynamic microtubules have been shown to enter dendritic spines, which correlates with NMDA receptor-dependent spine structural plasticity [42,44,45], an important future goal will be to determine whether NMDA receptor-mediated Par1 activation plays a role in this dynamic microtubule entry during spine plasticity.

Finally, it would be interesting to explore other potential upstream regulators of the LKB1/Par1 module at the synapse and any feedback regulations. It has been reported that LKB1 binds and phosphorylates AGS3/LGN, which affects the interaction of LGN with Gi proteins [46]. It will be important to examine whether LKB1 and Par1 are regulated by G protein-coupled receptors like mGluRs at the synapses. Conversely, because of the effects of LKB1 on LGN, it will be intriguing to see whether the LKB1/Par1 module regulates mGluR signaling at the synapses.

In summary, our studies show a novel mechanism by which the Ser/Thr kinase MARK/Par1 is activated at the neuronal synapse. We show that MARK/Par1 is activated downstream of NMDA receptors through a PKA-dependent mechanism. Our results suggest that MARK/Par1 is involved in activity-dependent synaptic plasticity. Further studies are needed to determine the downstream substrates of MARK/Par1 that are involved in this process.

## Supporting Information

S1 Fig Picrotoxin treatment increases Par1 activity. Hippocampal neurons at different stages (DIV11, 14, 17) were treated with 10μM picrotoxin for one hour, lysed and immunoblotted for phospho-Par1 (p-Par1), Par1 and α-tubulin.
(TIF)

S2 Fig Picrotoxin treatment increases Par1 activity in the synaptosomal fraction. DIV12 cortical neurons were treated with 4-AP and picrotoxin for 10 min. After treatment, the crude synaptosomal fraction was isolated and immunoblotted with the indicated antibodies.
(TIF)

S3 Fig Synaptic NMDA receptor stimulation activates Par1. Hippocampal neurons were stimulated with 4-AP and bicuculline for 10 min, lysed and immunoblotted with the indicated antibodies.
(TIF)

S4 Fig LKB1 activity shows a dose-dependent increase upon forskolin treatment. Hippocampal neurons were treated with various concentrations of forskolin, lysed and immunoblotted with the indicated antibodies.
(TIF)

S5 Fig Activated LKB1 is present at excitatory synapses. Hippocampal neurons (DIV24) were fixed and permeabilized with MeOH and immunostained with phospho-LKB1 (p-LKB1) and PSD-95 antibodies. Arrows point to colocalization.
(TIF)
