# Supplementary figures and images for: MARK/Par1 Kinase Is Activated Downstream of NMDA Receptors through a PKA-Dependent Mechanism
Source: PLoS One. 2015 May 1;10(5):e0124816. doi: 10.1371/journal.pone.0124816 (PMC4416788; doi:10.1371/journal.pone.0124816)

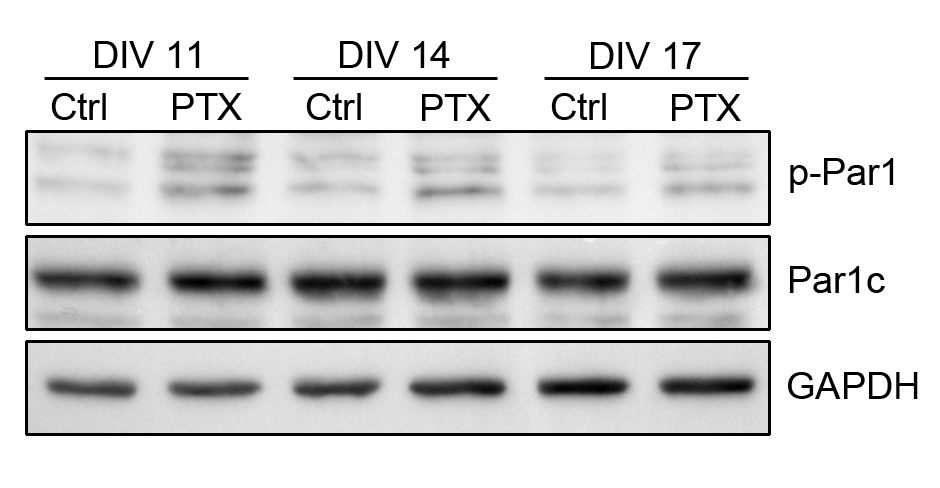

Supplement: S1 Fig — Hippocampal neurons at different stages (DIV11, 14, 17) were treated with 10μM picrotoxin for one hour, lysed and immunoblotted for phospho-Par1 (p-Par1), Par1 and α-tubulin. (TIF) [file pone.0124816.s001.tif]

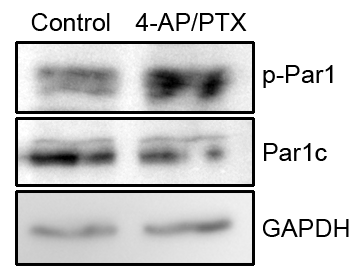

Supplement: S2 Fig — DIV12 cortical neurons were treated with 4-AP and picrotoxin for 10 min. After treatment, the crude synaptosomal fraction was isolated and immunoblotted with the indicated antibodies. (TIF) [file pone.0124816.s002.tif]

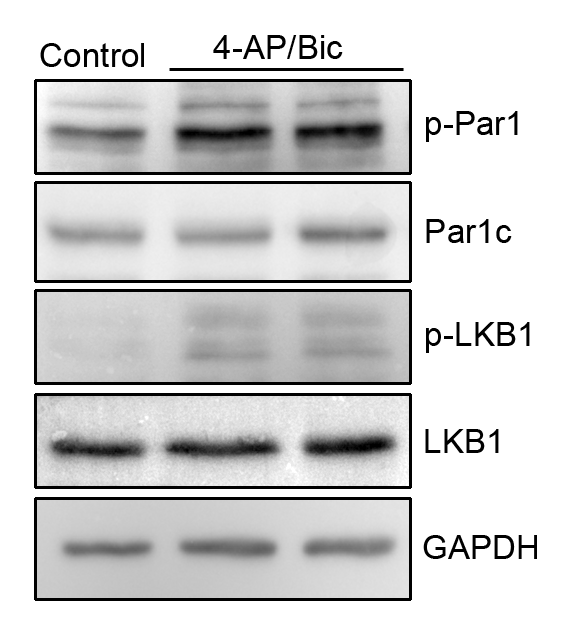

Supplement: S3 Fig — Hippocampal neurons were stimulated with 4-AP and bicuculline for 10 min, lysed and immunoblotted with the indicated antibodies. (TIF) [file pone.0124816.s003.tif]

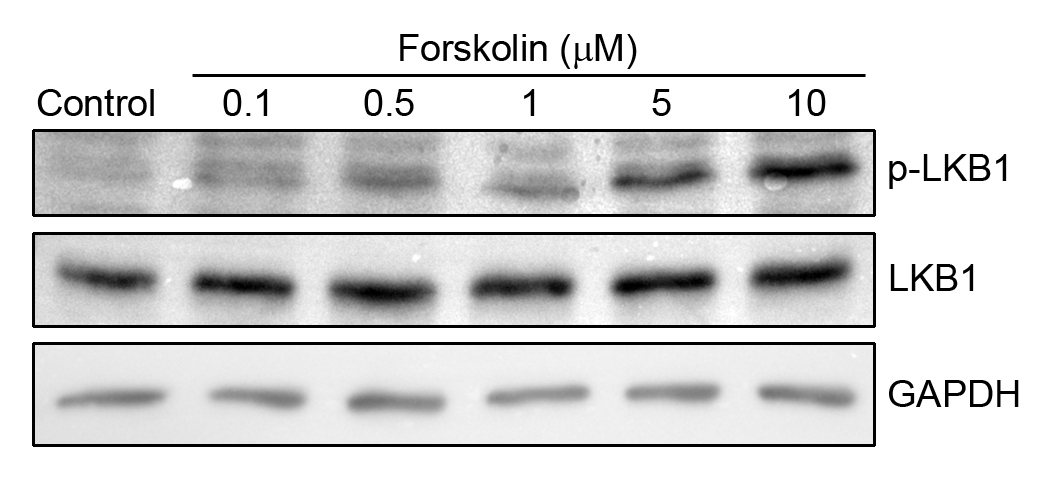

Supplement: S4 Fig — Hippocampal neurons were treated with various concentrations of forskolin, lysed and immunoblotted with the indicated antibodies. (TIF) [file pone.0124816.s004.tif]

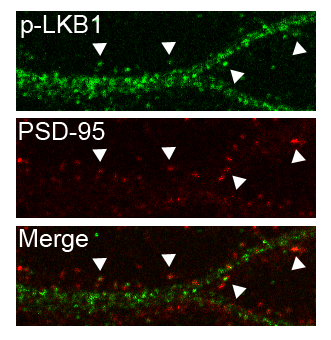

Supplement: S5 Fig — Hippocampal neurons (DIV24) were fixed and permeabilized with MeOH and immunostained with phospho-LKB1 (p-LKB1) and PSD-95 antibodies. Arrows point to colocalization. (TIF) [file pone.0124816.s005.tif]
